# Managing Comorbid Eating Disorders and Autism Spectrum Conditions: An Eden Unit Quality Improvement Project

**DOI:** 10.1192/bjo.2024.419

**Published:** 2024-08-01

**Authors:** Dharmesh Rai, Lily Davida

**Affiliations:** NHS Grampian, Aberdeen, United Kingdom

## Abstract

**Aims:**

Currently, there is an absence of clear guidelines or recommendations for individuals with an eating disorder (ED) and comorbid autism spectrum conditions (ASC).

The Maudsley ED team has pioneered a tailored approach for comorbid ED and ASC called the PEACE pathway.

Our aim is to adapt and implement a similar pathway within the Eden Unit (NHS Grampian inpatient eating disorder service).

**Methods:**

Questionnaires targeted two key stakeholders: patients and staff.

Patient questionnaires had 18 multiple-choice questions on a Likert scale, along with space for comments. The questions aimed to assess inpatient care adequacy in terms of care, routine, environment, mealtimes, and staff members. There were also specific questions related to ASC, examining whether sensory and communicative needs are being met and taken into account.

Staff questionnaires had 10 ‘yes-or-no' questions, along with space for comments, and gauged attitudes toward managing comorbid ED and ASC.

**Results:**

6/7 patient questionnaires were completed. 3 patients have comorbid ASC.

One patient found the ward overwhelming due to ASC, while others found it suitable. All experienced distress transitioning from outpatient to inpatient services, with subsequent admissions proving less challenging when they knew what to expect. They were allowed to have safe sensory items e.g comfort toys, headphones etc.

Generally, they felt well-supported during distress and felt their communication needs were met by nurses and HCSWs but not always by dietitians and clinicians due to a lack of availability. Some were frustrated with vague menu descriptions and there was some diagnostic overshadowing over dislike of certain foods.

13/20 staff questionnaires were completed. It showed most staff did not have formal training in managing comorbid ASC and ED, and confidence and skills varied in proportion to time and experience in the service. All staff members expressed they would like formal training, through sessions such as monthly training, weekly huddles, or psychoeducation.

**Conclusion:**

A significant proportion of inpatients have comorbid ED and ASC. Therefore, awareness of potentially greater needs around communication, environment, and sensory hyper- or hyposensitivity is important. There is a risk of diagnostic overshadowing as both ED and ASC can mimic similar symptoms: cognitive rigidity, fixation on certain things etc. So while not straightforward it is important to differentiate which symptoms are due to ASC and which are due to ED. Leveraging resources from the PEACE pathway website, both staff and patients can enhance their understanding of this complex comorbidity.

